# Fecal and soil microbiota composition of gardening and non-gardening families

**DOI:** 10.1038/s41598-022-05387-5

**Published:** 2022-01-31

**Authors:** Marina D. Brown, Leila M. Shinn, Ginger Reeser, Matthew Browning, Andiara Schwingel, Naiman A. Khan, Hannah D. Holscher

**Affiliations:** 1 Department of Food Science and Human Nutrition, University of Illinois Urbana-Champaign, Urbana, IL USA; 2 Division of Nutritional Sciences, University of Illinois Urbana-Champaign, Urbana, IL USA; 3 Department of Kinesiology and Community Health, University of Illinois Urbana-Champaign, Urbana, IL USA; 4 Parks, Recreation, and Tourism Management, Clemson University, Clemson, SC USA; 5 Family Resiliency Center, University of Illinois, Urbana, IL USA

**Keywords:** Urban ecology, Nutrition, Microbiota

## Abstract

Historically, humans have interacted with soils, which contain a rich source of microorganisms. Fruit and vegetable gardening is the primary interaction humans have with soil today. Animal research reveals that soil microorganisms can be transferred to the rodent intestine. However, studies on fecal and soil microbial changes associated with gardening in humans are lacking. The current case-controlled cohort study aimed to characterize the fecal and soil microbiota of gardening families (n = 10) and non-gardening (control) families (n = 9). Families included two adults and one child (5–18 years) for a total of 56 participants. All participants provided a fecal sample, soil sample, and diet history questionnaires before the gardening season (April) and during the peak of the gardening season (August). Healthy Eating Index (HEI-2015) scores and nutrient analysis were performed. Fecal and soil DNA were extracted and amplified. Sequence data were then processed and analyzed. Peak season gardening families tended to have greater fecal operational features, a greater Faith's Phylogenetic Diversity score, greater fiber intake, and higher abundances of fiber fermenting bacteria than peak control families. Soil endemic microbes were also shared with gardening participant’s fecal samples. This study revealed that the fecal microbiota of gardening families differs from non-gardening families, and that there are detectable changes in the fecal microbial community of gardeners and their family members over the course of the gardening season. Additional research is necessary to determine if changes induced by gardening on the gut microbiota contribute to human health.

## Introduction

Humans have a unique intestinal microbial composition that can be influenced and differentiated by genetics and lifestyle factors. The human gut is one of the most abundantly populated microbial environments^1^. The gut microbiota affects host digestion, metabolism, and immunity^2,3^^.^ Disruption of gut microbial homeostasis may contribute to the development of non-communicable diseases, including obesity and inflammatory bowel disease^4–7^. Westernized diet, medicine, and lifestyle patterns, such as increased hygienic practices, are hypothesized to underlie microbiota disruptions as well as the growing prevalence of non-communicable diseases^8,9^. Therefore, it is critical to study how the gut microbiota is changing in urban regions and to understand the factors that influence gut bacteria composition as they may inform our understanding of links between environmental factors and health.

Genetics partially inform the human gut microbiota; however, environmental interactions drive diversity and colonization in the gut^10^. Genetically similar individuals who co-habitat (e.g., families) have unique microbial makeups while sharing gut microbes with their housemates^11^ and skin microbes with their pets^12^. Preclinical evidence shows that environmental habitat exposures can change fecal microbial communities^13^. For example, mice exposed to soil, house dust, and decaying plant materials experienced an increase in alpha diversity, which correlated with lower serum IgE concentrations, indicating a reduced autoimmune response^14^.

Humans evolved with environmental microorganisms through exposure to soil, historically through hunting and gathering activities^15^ or living on traditional farms^16,17^. The transition from hunting and gathering to domesticated agriculture approximately 10,000 years ago is thought to have contributed to the dramatic change in gut microbial composition in humans^18^. Indeed, many rural societies have higher fecal bacteria richness when compared to Western populations^15,19,20^ and different environmental exposures could in part dictate these microbial differences.

Diet is another important environmental factor that affects gut microbial composition^21^. Foods like fruits, vegetables, whole grains, nuts, and legumes contain dietary fibers that can be metabolized by intestinal microorganisms^22^. Greater intake of plants, including fruits and vegetables, is also linked with greater gut microbiota diversity^23^. Non-industrialized communities with traditional lifestyles have more intestinal bacteria with a higher capacity to ferment complex carbohydrates when compared to modernized Italians^15^. Dietary differences between these groups likely contribute to these differences as traditional communities consume higher amounts of fiber than urban residents^15,19^.

It has been suggested that reintroducing humans to urban green spaces can alter gut microbiota composition to potentially reduce diseases linked with urbanization^24^. Gardening provides exposure to green spaces and environmental microorganisms, as well as the production of fibrous foods. However, research on gardening and the human gut microbiota is lacking. To address this gap, we designed a study with the primary objective of investigating gut microbiota differences between gardening families (> 30 min of gardening per week) and their non-gardening counterparts. Further, this research aimed to determine if gardeners would eat more fruits and vegetables, leading to a higher diet quality than non-gardeners by peak garden season. We also aimed to understand if soil bacteria were present in the fecal microbiota of gardeners. We hypothesized that the expected increase of diet quality in conjunction with direct soil interaction would lead to changes in the gut microbiota of routine gardeners and their family members.

## Results

### Dataset characteristics

A total of ten gardening family units (n = 30 individuals) and nine control family units (n = 27 individuals) participated. Family units consisted of three members living within the same household, including a primary adult gardener, another adult member living in the same household, and a child between ages 5 and 18. The child and corresponding adult may have assisted the primary gardener with gardening activities. Participant demographics are listed in Table 1. Baseline data was collected before the gardening season began (April). Peak gardening season (August) constituted as when most produce could be readily harvested and consumed.

**Table 1:** Participant demographics.

Characteristic^1^	Control (n)	Gardeners (n)
**Total participants**	27	30
Adult Participants	18	20
Child Participants	9	10
**Adult age (years)**		
19–29	0	1
30–39	7	9
40–49	9	9
$\ge$ 50	1	1
**Child age (years)**		
5–8	4	3
9–12	4	5
13–18	1	2
**Adult level of education**		
High School	1	0
Some College	3	1
College Degree	5	8
Advanced Degree	6	10
**Participant ethnicity**		
Asian	3	0
Black or African American	2	0
Native Hawaiian or Pacific Islander	0	0
White or Caucasian	16	27
Mixed or Other	2	3

^1^All characteristics reported as n.

**Figure 1:**
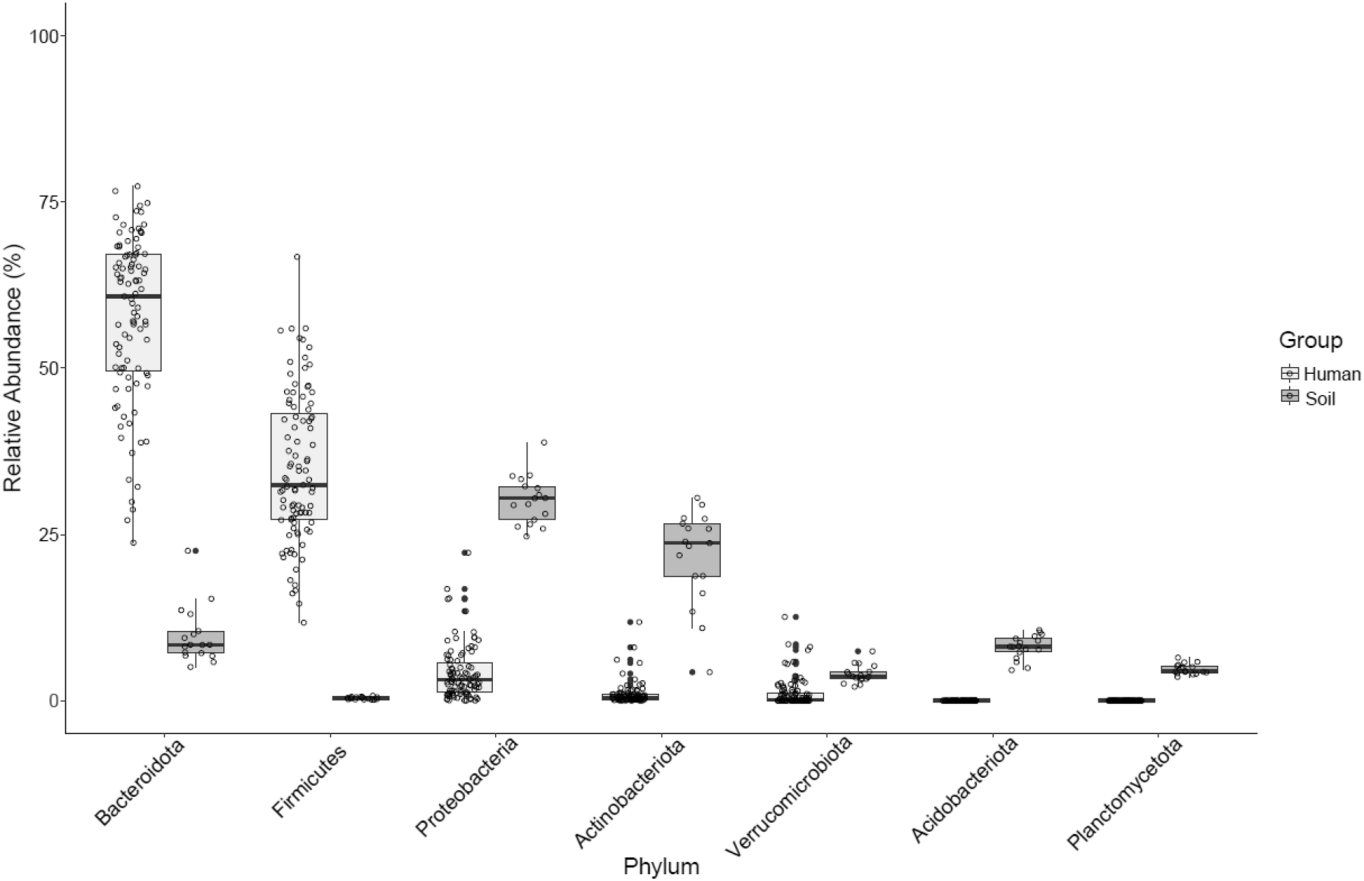
Average relative abundance of dominant phyla found within human and soil samples.

After demultiplexing and quality control, the mean amplicon sequence variant (ASV) in the human fecal samples was 124,865 $\pm$ 3,259, with a total ASV frequency of 11,862,265. ASV per sample included a minimum of 26,639 counts, where we rarified for diversity metrics accordingly, and a maximum of 201,068 counts. A total of 3,329 ASVs were found. At the phyla level, Bacteroidota dominated human gut samples with an average abundance of 58%, followed by Firmicutes 35%, and Proteobacteria 4%. The average ASV count in the soil samples (n = 17) was 122,002 $\pm$ 8,164 with a total ASV frequency of 2,074,035. ASV per soil sample ranged from 78,342 to 197,789, with a total of 20,416 features found. Samples were dominated at the phyla level by Proteobacteria 30%, Actinobacteria 22%, and Bacteroidetes at 9.8%. Comparisons of the major phyla found in human and soil samples are in Fig. 1.

### Fecal microbiota differences between gardening and non-gardening families

Alpha diversity metrics of fecal samples were analyzed between control and garden participants using QIIME2 (v. 2020.6). These metrics included a total count of observed ASV features and Faith's Phylogenic Diversity (Faith's PD).

**Figure 2:**
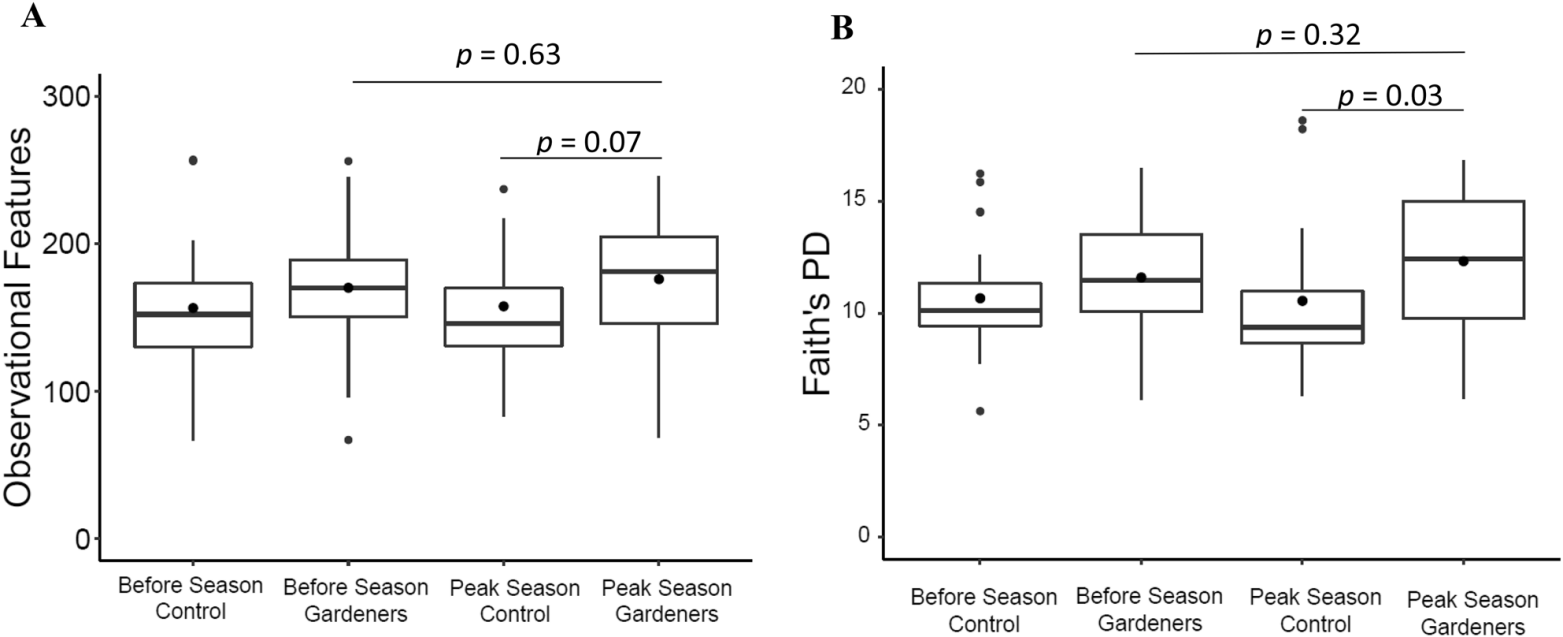
Comparisons of alpha diversity metrics including (**A**) Operational Features and (**B**) Faith’s Phylogenic Diversity between all participants across the gardening season. Kruskal–Wallis pairwise statistics were used to test differences.

Gardening families tended to have more observed ASV features (175.2 $\pm$ 43.6 vs. 157.4 $\pm$ 53.3; p = 0.07) (Fig. 2A) and higher Faith's PD scores (12.3 $\pm$ 3.06 vs. 10.5 $\pm$ 3.13; p = 0.03) (Fig. 2B) at peak gardening season (hereafter, "peak season") than peak control families. Gardening families tended to have more observed features before the gardening season (169.8 $\pm$ 45.5; p = 0.08) than control families at peak season. Moreover, beta diversity analysis including unweighted UniFrac (p = 0.07), Bray Curtis (p = 0.02) and Jaccard (p = 0.01) distances varied between gardening families at peak season compared to control family distances at peak season (Supplementary Figure S1).

**Figure 3:**
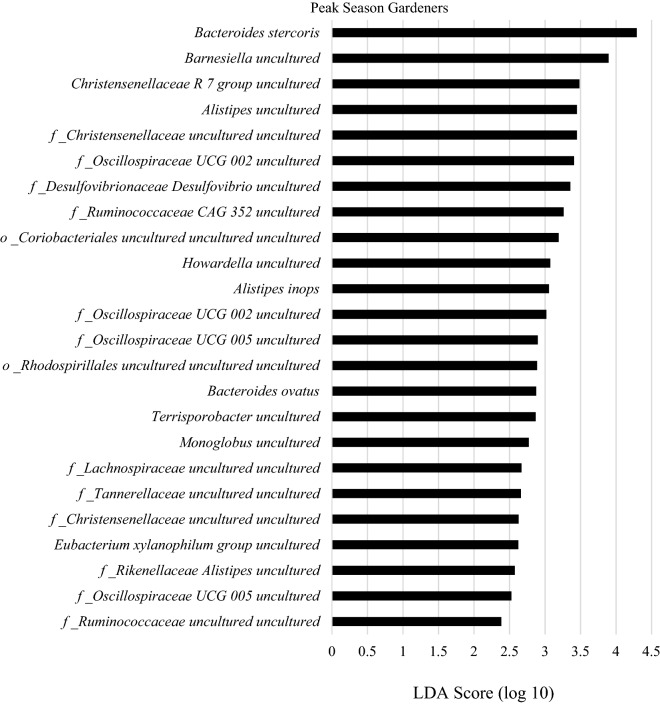
LEfSe analysis at the species level between control and garden families at peak gardening season. Microbes shown are significantly greater in peak season gardening families.

Linear discriminate analysis effect size (LEfSe) of gardening families and control families at peak season revealed that gardening families had higher abundances of several gut microbial species (Fig. 3). Relative abundances of these taxa are reported in Table 2. Genus LEfSe scores of gardening families are shown in Supplementary Figure S2 with relative abundances in Supplementary Table S1. LEfSe species and genus abundances in control families are in Supplementary Figure S3 and Supplementary Figure S4 with abundances in Supplementary Table S2 and Supplementary Table S3.

**Table 2:** Significance of species greatest in gardeners at peak season when compared to peak season control.

Species enriched in peak season gardeners						
Species^1^	Control (%)	$\pm$ SEM	Garden (%)	$\pm$ SEM	q value^2^	p value^3^
*Eubacterium xylanophilum group uncultured*	0.00	0.00	0.03	0.01	0.02	0.00
*Bacteroides ovatus*	0.00	0.00	0.15	0.06	0.02	0.00
*Barnesiella uncultured*	0.62	0.28	2.04	0.49	0.05	0.00
*Alistipes inops*	0.00	0.00	0.22	0.09	0.05	0.01
*Alistipes uncultured*	0.00	0.00	0.60	0.30	0.05	0.01
*Alistipes uncultured*	0.07	0.04	0.09	0.02	0.05	0.01
*f_Christensenellaceae uncultured uncultured*	0.02	0.00	0.04	0.00	0.05	0.01
*f_Christensenellaceae R 7 group uncultured*	0.19	0.11	0.68	0.51	0.05	0.02
*Desulfovibrio uncultured*	0.00	0.00	0.41	0.18	0.05	0.02
*f_Christensenellaceae uncultured uncultured*	0.00	0.00	0.001	0.00	0.05	0.02
*f_Tannerellaceae uncultured uncultured*	0.00	0.00	0.07	0.04	0.05	0.02
*Monoglobus uncultured*	0.00	0.00	0.06	0.05	0.05	0.03
*f_Oscillospiraceae UC* 002 *uncultured*	0.38	0.18	0.89	0.44	0.05	0.04
*Bacteroides stercoris*	2.62	1.20	6.24	1.69	0.05	0.04
*f_Ruminococcaceae CA* 352 *uncultured*	0.41	0.15	0.75	0.17	0.05	0.04
*f_Ruminococcaceae uncultured uncultured*	0.05	0.04	0.08	0.03	0.05	0.04
*f_Oscillospiraceae UC* 005 *uncultured*	0.00	0.00	0.01	0.00	0.05	0.04
*f_Lachnospiraceae uncultured uncultured*	0.03	0.00	0.05	0.00	0.05	0.04
*f_Oscillospiraceae UC* 002 *uncultured*	0.09	0.03	0.30	0.10	0.05	0.04
*f_Oscillospiraceae UC* 005 *uncultured*	0.09	0.03	0.14	0.03	0.05	0.04
*Howardella uncultured*	0.00	0.00	0.01	0.00	0.05	0.04
*o_Rhodospirillales uncultured uncultured uncultured*	0.00	0.00	0.12	0.07	0.05	0.04
*o_Coriobacteriales uncultured uncultured uncultured*	0.00	0.00	0.002	0.00	0.05	0.04
*Terrisporobacter uncultured*	0.00	0.01	0.02	0.00	0.05	0.05

^1^Average relative abundances (%) of significant species ± SEM.

^2^False Discovery Rate was used for adjustment.

^3^Wilcoxon Rank Sum Test.

After p-value adjustment for multiple comparisons, *Bacteroides* *ovatus* (p = 0.02) and *Eubacterium xylanophilum group spp.* (p = 0.02) abundances were greater in gardening families than in control families at peak season, although at low abundances (Table 2). The abundance of class level *Alphaproteobacteria* tended to be 64% higher in gardening families than control families at peak season (p = 0.06).

Gardening families also had higher abundances of unassigned taxa. Specifically, gardening families had more unassigned taxonomic genera before the gardening season (2.8%) and at peak season (3.9%) than control families (baseline: 2.0%, peak: 2.7%).

### Fecal microbiota differences within gardening families across the gardening season

**Figure 4:**
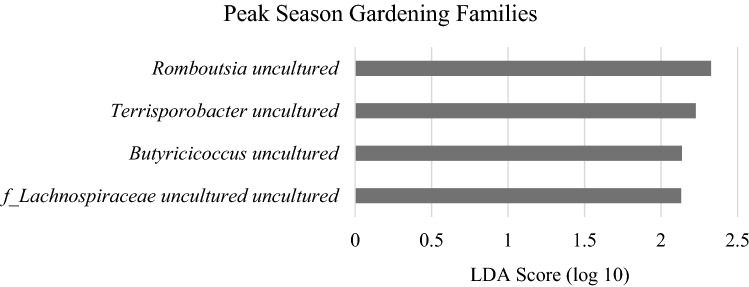
LEfSe analysis of species level in gardening families before the garden season and at peak garden season. Species shown are significantly greater at peak gardening season than before the garden season.

**Figure 5:**
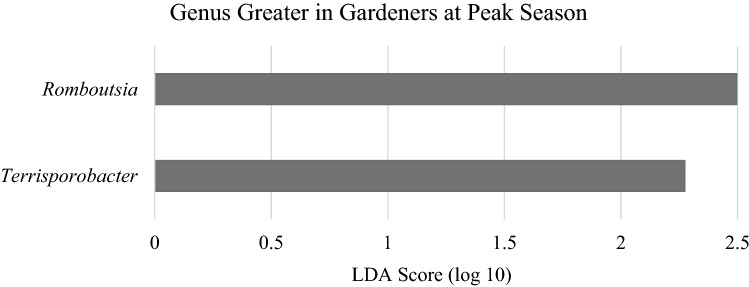
LEfSe analysis of the genus level in gardeners before the garden season and at peak garden season. Genus shown are significantly greater at peak gardening season than before the garden season.

Within gardening family fecal samples, alpha diversity metrics numerically increased from before the gardening season to peak season but were not statistically significant. These metrics included observational features (baseline: 157 $\pm$ 45.0, peak: 175 $\pm$ 43.6; p = 0.6) (Fig. 2A) and Faith’s PD (baseline: 11.5 $\pm$ 2.7, peak: 12.3 $\pm$ 3.0; p = 0.3) (Fig. 2B). Beta diversity distance metrics were not different across the gardening season in gardening families, including weighted (p = 0.98) and unweighted UniFrac (p = 0.99), Bray–Curtis (p = 0.88), and Jaccard (p = 0.96) (Supplementary Figure S5). At the species level, LEfSe analysis revealed that gardening families had significantly greater abundances of *Romboutsia uncultured, Terrisporobacter uncultured, Butyricicoccus uncultured,* an uncultured genus in the *Lachnospiraceae* family at peak season than before the gardening season (Fig. 4). Over this time period, *Romboutsia* increased 77% (p = 0.03) and *Terrisporobacter* increased sixfold (p = 0.01) (Fig. 5).

**Figure 6:**
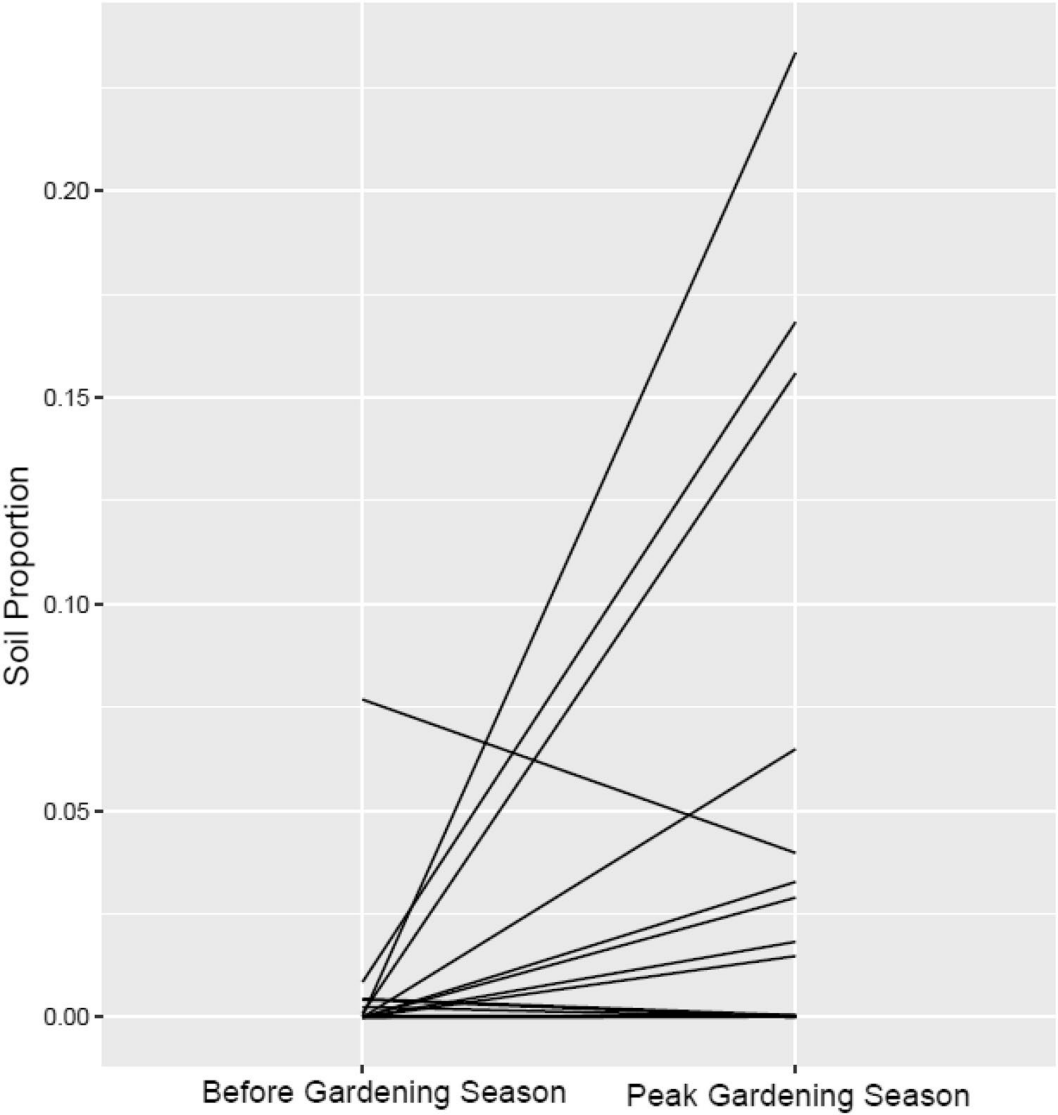
Estimations from FEAST output on contribution of soil (source) microbes to the gut (sink) of gardening families from before the garden season to peak garden season. Lines represent the gain or loss of soil endemic microbes during the gardening season.

Fast expectation–maximization for microbial source tracking (FEAST)^25^ was utilized to investigate the potential origin of fecal microbes by using respective gardening plot soil samples as sources of taxa. Results revealed a contribution of soil endemic microbes into the gut of gardening families, suggesting a taxon movement (Fig. 6). Average proportions of shared soil microbiota increased across the gardening season (p = 0.03). Eight of the nine gardening families reported gardening with their children (Supplementary Table S4). Children fecal samples had the highest proportions of these soil microbiota at peak season (children: 7.6% $\pm$ 3.3, adults: 3.2% $\pm$ 1.7; p = 0.47).

The highest proportion of soil bacteria found in a gardening family member at peak season was 23%, which had increased from a proportion of 0.02% before the gardening season. This participant's child also had an increase in soil bacteria in their gut from 0.09% before the gardening season to 15% at peak season. This family reported gardening at least 5–8 h per week, gardening with their child, and eating their harvested produce almost every day.

### Dietary differences between gardening and non-gardening families

**Figure 7:**
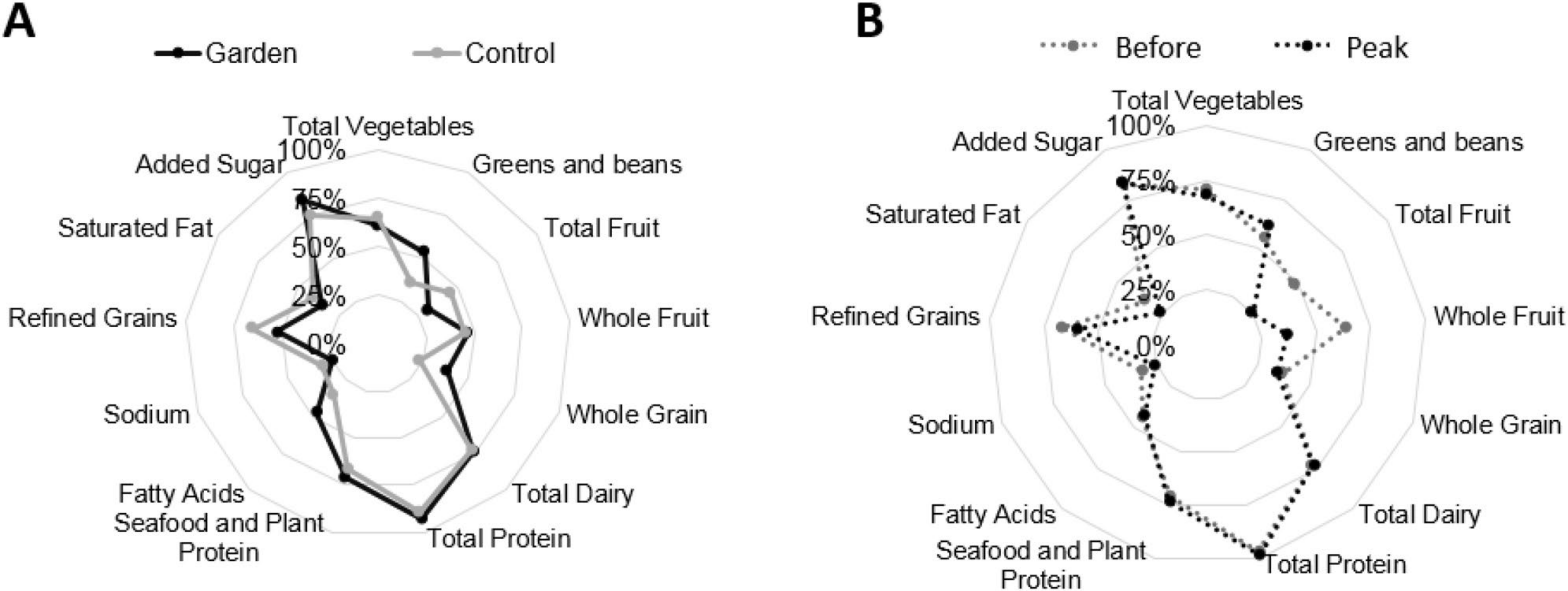
Radar graphs of average HEI scores between members of (**A**) the gardening and control families at peak gardening season and (**B**) within the gardening families from before the gardening season to peak gardening season.

Nutrient level analyses generated from self-reported dietary data and corresponding statistics are reported in Tables 3 and 4. Gardening families' vitamin C intake at peak season was 67% greater than control families' intake at peak season. Similarly, gardening families' vitamin K intake was 27% greater than control families' intake at peak season. Iron and selenium intakes were also different; gardening families reported 24% more iron intake and 22% more selenium intake at peak season than peak control families. Folate intake was also 31% higher in gardening families than non-gardening families at peak season. Average total fiber intake was 19% higher in gardening families than in control families at peak season. Average calculated HEI-2015 total scores at peak season were not different between gardening families (53.5 $\pm$ 10.9) and control families (52.9 $\pm$ 9.8) (p = 0.85) (Fig. 7A).

**Table 3:** Diet record nutrient differences at peak gardening season between gardening and control families.

Nutrient^1^	Peak season control		Peak season gardeners		p value^2^
	Average	SEM	Average	SEM	
Vitamin C (mg)	48.2	3.81	75.4	5.8	0.00
Vitamin K (mcg)	106	10.7	130	12.3	0.03
Folate (mcg)	264	18.9	348	26.7	0.03
Iron (mg)	13.1	0.6	16.3	0.9	0.00
Selenium (mcg)	109	4.8	123	5.6	0.09
Fiber (g)	14.5	0.7	17.3	0.8	0.00

^1^Average nutrient values $\pm$ SEM.

^2^Two-sided Wilcoxon test.

**Table 4:** Diet record nutrient differences within gardening families from before the garden season to peak garden season.

Nutrient^1^	Gardening families				
	Before garden season		Peak garden season		p value^2^
	Average	SEM	Average	SEM	
Potassium (mcg)	2350	83.7	2050	111	0.01
Folate (mcg)	419	21.0	348	26.7	0.002
Selenium (mcg)	101	43.1	123	5.6	0.005

^1^Average nutrient values $\pm$ SEM.

^2^Two-sided Wilcoxon test.

### Dietary differences within gardening families across the gardening season

Selenium increased by 22% in gardeners from before the gardening season to peak season. In contrast, gardening families' potassium intake was 12% lower at peak season than before the gardening season. Folate intake decreased by 17% in gardening families from before the gardening season to peak season. Gardening families had a 41% (p = 0.04) reduction in total fruit and a 30% (p = 0.04) reduction in whole fruit HEI-2015 component scores from before the gardening season to peak season (Fig. 7B).

## Discussion

Gardeners represent a group of urbanized humans that regularly interact with a soil environment. Therefore, this study aimed to characterize the human fecal and soil microbiota to understand the bacterial relationships between these two environments. Our results revealed that gardening families' fecal samples tended to have a higher alpha diversity at peak garden season compared to peak control. Although at lower abundances, specific fecal bacteria that were higher among gardening families at peak season included *Bacteroides ovatus,* an uncultured *Eubacterium xylanophilum group spp.*, and *Bacteroides stercoris* when compared to peak control. Self-reported dietary intake of total fiber, iron, selenium, vitamin C, and vitamin K levels were higher in gardening families than control families at peak season with selenium increasing within gardening families throughout the gardening season.

Most gardening families in our study reported growing fruits and vegetables while eating their produce at least once a week. The gardening group tended to have more ASVs when compared to control at peak season. A large cross-sectional study of over 10,000 adults reported that those that consumed more than thirty different plant foods per week had higher fecal observed OTU counts than individuals who reported eating less than ten different plant foods per week^23^. Intervention studies have demonstrated that consuming specific plant foods daily (e.g., almonds and avocados) results in higher fecal microbiota diversity scores in adults^26,27^. Furthermore, higher long term cumulative and recent healthy diet scores in Chinese adults were associated with higher alpha diversity scores^28^. Higher recent healthy diet scores were also associated with abundances of unknown species of fecal microbes in this population^28^. As our results are based on longitudinal assessments, causality cannot be determined from this study. Thus, intervention studies that prospectively assign individuals to gardening or control groups are needed to determine how diet and gardening can affect alpha diversity outcomes.

Total average fiber intake was greater in gardening families than non-gardening families at peak season. Correspondingly, gardening families’ fecal samples had greater abundances of several fiber fermenting bacteria at peak season when compared to control families at peak season. Fruits and vegetables contain varying amounts of non-digestible fibers, which can be fermented and metabolized by intestinal microbes^29^. Our results revealed that *Bacteroides ovatus* and *Bacteroides stercoris* were greater in the fecal microbiota of gardening families at peak season. Others have reported that *Bacteroides ovatus* was enriched in a pectin fermentation group after an in vitro investigation of the effect of non-digestible fibers on fecal microbial composition, demonstrating its fiber fermenting abilities^30^. Further, *Bacteroides* was enriched in adults after a complete feeding broccoli intervention when compared to control^31^.

Our results also revealed that uncultured *Butyricicoccus spp.* was higher in gardening families' fecal samples at peak gardening season when compared to before gardening season. Further, uncultured *Eubacterium xylanophilum group spp.* was greater, although at low abundances, in gardening families than in control families at peak gardening season. Genus *Eubacterium* is a butyrate producer^30^ with some species having the ability to ferment pectin^32^. An enrichment of genuses *Butyricicoccus* and *Eubacterium* have previously been reported in the Hadza hunter-gatherer community^15^ with *Eubacterium* being enriched in an Irish Traveller subgroup^33^ and at significantly higher proportions in a rural Papua New Guinea population^20^. Irish Travellers are a nomadic group residing in Europe that diverged from its settled community approximately 200 to 1,200 years ago^33^. They were recently forced into a lifestyle change with restricted land access that essentially ended their nomadism. Indigenous, cave-dwelling communities and the Irish Traveller group have similar gut microbial structures^33^. Indigenous communities consume high amounts of fiber^15,19^, whereas the Irish Traveller group consumed the least amount of fiber when compared to industrialized groups^33^. A similar gut microbial composition suggests these indigenous and nomadic communities have similar lifestyles, but that their microbial contributions could be independent of diet.

We were interested in investigating the potential transmission of microbes from garden soil to the human gut. Our results revealed that soil-derived bacteria were detected in the human fecal samples and increased with direct and indirect soil contact. Even though there was only one primary gardener per family unit in this study, the sharing of soil-sourced bacteria was detected in most (20 of 23) gardening individuals. However, one individual did experience a decrease of soil derived microbes over the course of the gardening season. Gardening families' fecal samples had a total of 24 primarily unassigned species that were enriched at peak season when compared to non-gardening, control families. Soil is a highly variable environment consisting of many low-abundance taxa and, due to this diversity, many soil taxa remain unidentified^34^. Preclinical studies have reported the opportunistic nature of soil-dwelling microbes leading to gut colonization and diversity in mice^13,14,35^. Seedorf et al. tracked changes in gut microbiota composition by exposing mice to microbes of different environments^13^. It was reported that a single soil-derived bacterium reached an abundance of 57% in a germ-free bystander and its three cagemates and persisted, albeit at lower abundances, for a total of 21 days^13^. Schnorr compared soil OTUs with host-microbial DNA to find soil-endemic bacteria incorporated into the gut microbiome of various hosts, including humans with different lifestyle patterns^17^. Previous research has found that sharing a living space has a greater impact on gut microbial composition similarity than genetic relationships^10,36^. Since some primary gardeners reported gardening with their spouse and/or child, this shared activity combined with sharing a household is likely how most gardening participants aquired a proportion of soil microbes in their gut by the end of the gardening season.

The soil microbiome is one of the most diverse environments studied. Consistent contact with a respective gardening plot for the duration of the growing season may have resulted in a transmission of soil-endemic microbes, which concluded with a more diverse gut microbial structure and a different bacterial composition in our gardening participants. Research associated with gardening and soil microbial interactions is lacking. Changes in gut microbiota composition in urbanized regions have been linked to the rise of inflammatory and non-communicable diseases^37^. Evidence suggests that fibrous diets^22^ and environmental interactions^38^ can increase exposure to beneficial bacteria and potentially mediate this disease prevalence. Gardening remains the primary source of soil contact in the modern era and provides access to fibrous fruits and vegetables. Although the findings reported herein give insight into how gardening is associated with the fecal microbiota, further work is necessary to determine if connections between gardening and the gut microbiota are also linked to health outcomes.

Several limitations of the present work should be acknowledged. Firstly, the longitudinal design of the present study precludes the ability to determine causality. Thus, longitudinal studies with a larger sample population and intervention studies are necessary to understand connections between diet, gardening, and fecal microbiota. Additional work is also needed to determine how changes in the microbiota may be connected to health benefits associated with gardening. Further, intervention studies would help to address potential confounding factors, such as psychological or social-economic effects (i.e. poor access to garden space, affordability of healthy foods) that can play a role in healthy eating, microbial outcomes, and health status. Also, we acknowledge that many of the microbes that were greater in gardening families were present in low abundances, which may limit their biological relevance. An additional limitation of the current study is the lack of genus and species-level resolution of soil microbes due to its highly diverse environment.

This preliminary case-controlled cohort study reports on associations between gardening and the fecal and soil microbiota. Thus, future work should include assessment of health outcomes and additional characterization of the environmental and biological samples within larger cohorts and within intervention studies. For example, closer investigation of soil samples, including nutrient content, could inform how soil composition affects the health of gardeners. A collection of fecal fermentative end-products is also needed to better understand the interactions between diet and the gut microbiota of gardeners and non-gardeners. Future studies also should incorporate metagenomics sequencing or PCR to better characterize microorganisms and their functional capacities.

## Materials and methods

### Participants

A case-controlled cohort study was developed to compare differences in the fecal microbiota within families of habitual gardeners and control groups. Families were recruited from a survey-based study of gardeners and non-gardeners in the Urbana-Champaign, IL, area (Table 1). This study was approved by the University of Illinois Institutional Review Board and all methods were performed in accordance with relevant guidelines and regulations. Adult participants completed informed consent before participating in the study. Informed consent was obtained from a legally authorized individual or parents for minors participating in this study. All participants were examined before and after the gardening season and their fecal microbiota was compared to their non-gardening counterparts. Inclusion criteria consisted of having two adults and at least one child in a family of at least three individuals. Adults were included if they were between the ages of 19–55 years old. Child participants were required to be between the ages of 5 and 18. Within the gardening families, the primary gardener needed to garden at least 30 min a week for each week of the gardening season (April through August). Non-gardening families were identified as family units in which no member reported gardening. Participants were excluded if they were not living with their family members. Adult women were excluded if they were pregnant. Two participants were excluded from the human microbiota analyses based on poor sequencing depth.

Gardening families grew and consumed produce from their personal gardens while recording their dietary intake during the season. Participants completed a gardening survey before the gardening season and at peak gardening season to collect gardening habits and type of produce grown. Gardening family characteristics are shown in Supplementary Table S4. Produce that each family grew by peak garden season are listed in Supplementary Table S5. Questionnaires were given to participants to assess differences in demographics, family and individual activities, and health status. A general health questionnaire was administered before the gardening season and a gardening survey was distributed both before and at the peak of the gardening season.

### Sample collection

One fecal (n = 51) and one soil sample (n = 10) were collected before the gardening season (April 2018). Another set of samples (fecal n = 44, soil n = 7) were collected at the peak of the gardening season (August 2018). In total, 95 fecal samples and 17 soil samples were collected. Soil samples were retrieved from each gardening plot where the gardening family reported gardening. For soil samples, the soil surface was penetrated between 0 and 5 cm and 2.5 cm diameter to gather approximately 2 g of soil at each of ten locations in the garden.

### DNA Extraction and 16S rRNA sequencing

Once collected, fecal and soil samples were homogenized, processed, and stored at − 80 °C. DNA was extracted using the MoBio Powerlyzer Powersoil Kit (MoBIO Laboratories Inc., Carlsbad, CA, USA) by following the manufacturer's instructions. After extraction, the V4 region of 16S rRNA 515F (5'-GTGYCAGCMGCCGCGGTAA-3’) and 806R (5'-GGACTACNVGGGTWTCTAAT-3’) was amplified using a Fluidigm access array (Fluidigm, South San Francisco, CA, USA) and sequenced on a HiSeq (Illumina, Inc.)^39^ at the W.M. Keck Center for Biotechnology at the University of Illinois. This process allowed the retrieval of two reads at 250 nt each within a FASTQ file, as previously described^40^.

### Microbiota analysis

DNA sequences were analyzed in the form of a FASTQ file on QIIME2 2020.6.0^41^. FASTX-Toolkit (version 0.0.13) was used to trim primer sequences from the forward and reverse reads. The q2-dada2 plugin was used to filter sequences with a quality score $\ge$ 20. Forward and reverse reads were paired using q2-dada2 "denoise-paired" function^42^. The percentage of input reads that successfully merged was 90%. Only paired reads with a length of 253 base pairs were analyzed. Microbial taxonomy was assigned using the SILVA 138 database^43^. Alpha diversity metrics (observational features and Faith's PD) and beta diversity measures (Bray–Curtis, Jaccard, weighted UniFrac, and unweighted UniFrac distances) were assessed using QIIME2 core metrics.

### Diet records

Adult participants logged their daily food and beverage intake for five days before the gardening season and five days at peak gardening season using the Automated Self-Administered 24-Hour Dietary Recall (ASA24)^44^. Adults also completed a 5-day food journal for their child participants at these time points. These journals were analyzed using the Nutritional Data System for Research (NDSR)^45^. The HEI-2015 is a measure of diet quality using dietary components to create a total score out of 100. This score is used to test compliance with the U.S. Dietary Guidelines for Americans. The score reflects overall diet quality and the quality of each dietary component. HEI-2015 scores were calculated by obtaining the ratio of each dietary component to energy^45^. Ratios were then scored according to HEI-2015 scoring standards. The total component score is summed to calculate HEI-2015 total score. HEI-2015 component scores were calculated for all adult and child participants^46^.

### Statistical analysis

Statistical analyses were performed in R version 4.0.1 and Rstudio version 1.3.1093. Due to the exploratory nature of this research, P $\le$ 0.1 was considered a trend and P ≤ 0.05 was considered to be statistically significant for alpha- and beta-diversity analyses. To compare microbial abundances, taxa were transformed using a centered log ratio and a Wilcoxon test was utilized. Because many bacteria were assessed in this study, statistical analyses of bacterial abundances were adjusted with a false discovery rate^47^ with significance defined as q $\le$ 0.05 using the p.adjust function in R. LEfSe (Galaxy version 1.0) was also utilized to compare groups^48^. Statistically significant taxa were represented as having an alpha of ≤ 0.05 and a linear discriminant analysis (LDA) score of $\ge$ 2. An LDA score is a linear combination of continuous variables that characterizes or separates objects of interest. Fast expectation–maximization for microbial source tracking (FEAST)^25^ was used to determine the movement of microbes from the soil environment to the human gut. Gardening participants served as sinks and their corresponding soil sample was determined as source. The FEAST R package was used with default settings and a Boolean value of 1 to assign different sources per sink.

## Supplementary Information

Supplementary Information.

## Data Availability

Raw reads will be deposited into the NCBI Sequence Read Archive database under BioProject ID PRJNA741983.
