# Supplementary material for: Fecal and soil microbiota composition of gardening and non-gardening families
Source: Sci Rep. 2022 Jan 31;12:1595. doi: 10.1038/s41598-022-05387-5 (PMC8804003; doi:10.1038/s41598-022-05387-5)
Supplement: Supplementary file 1 — Supplementary Information. [file 41598_2022_5387_MOESM1_ESM.docx]

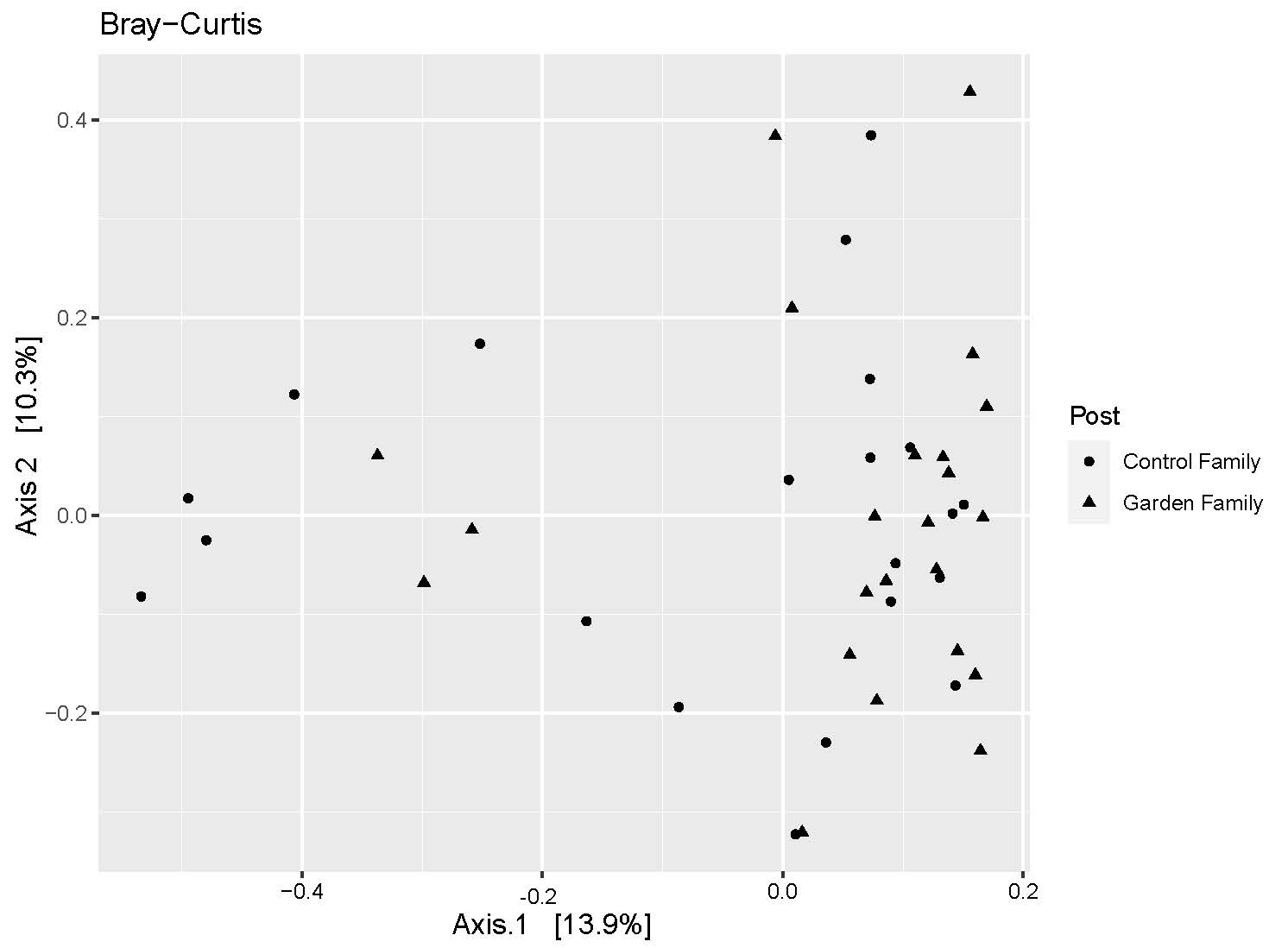

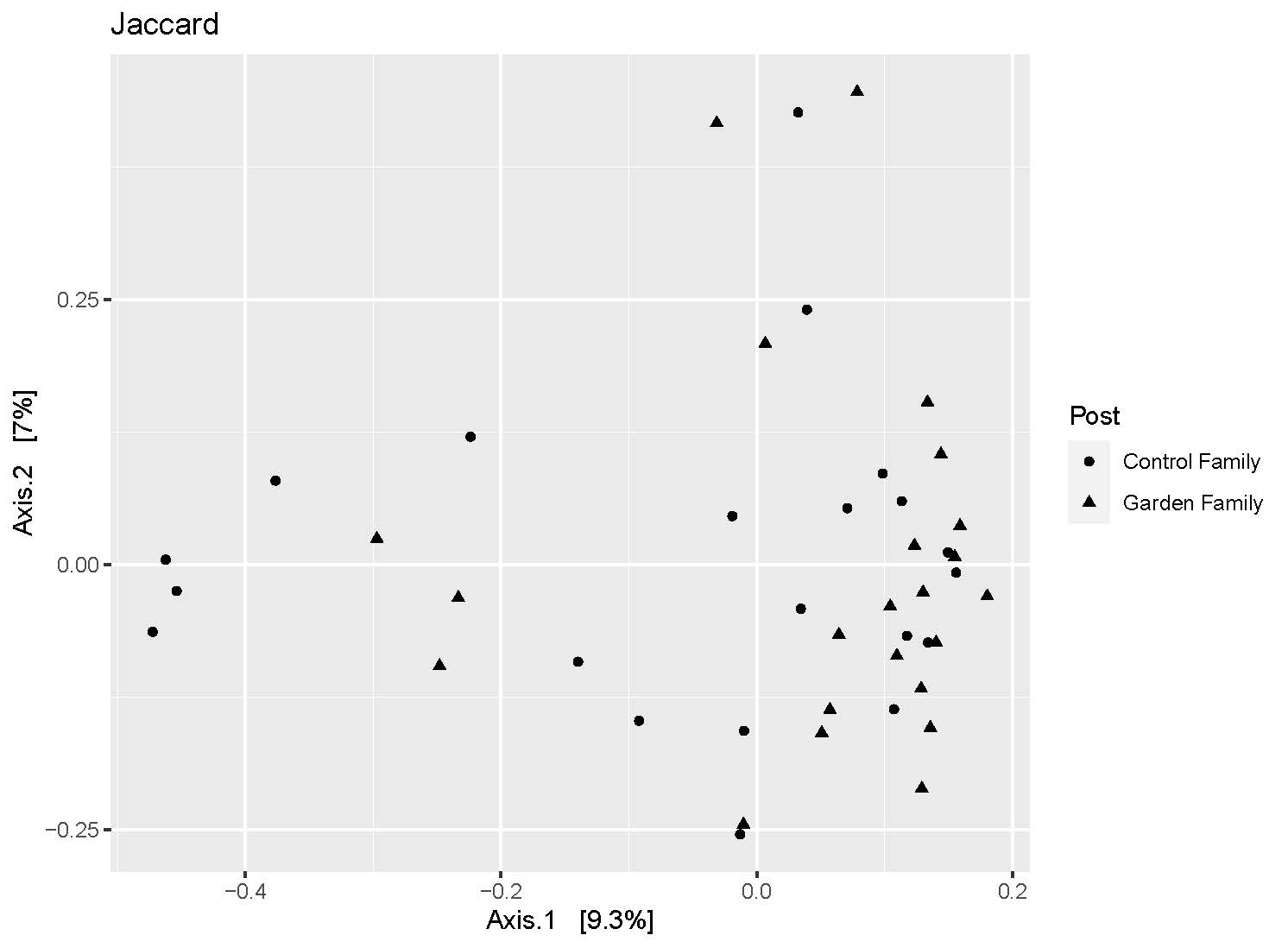


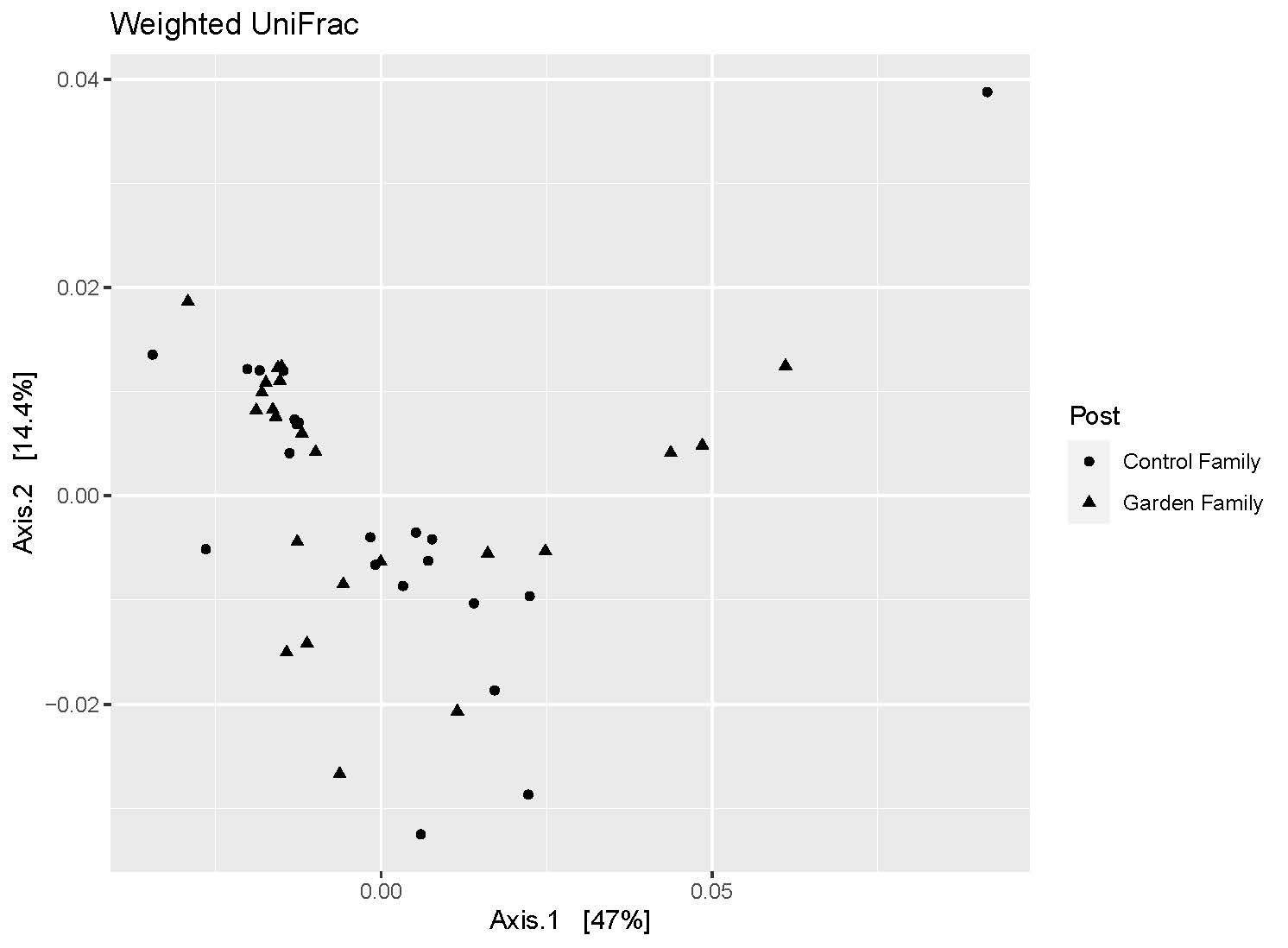

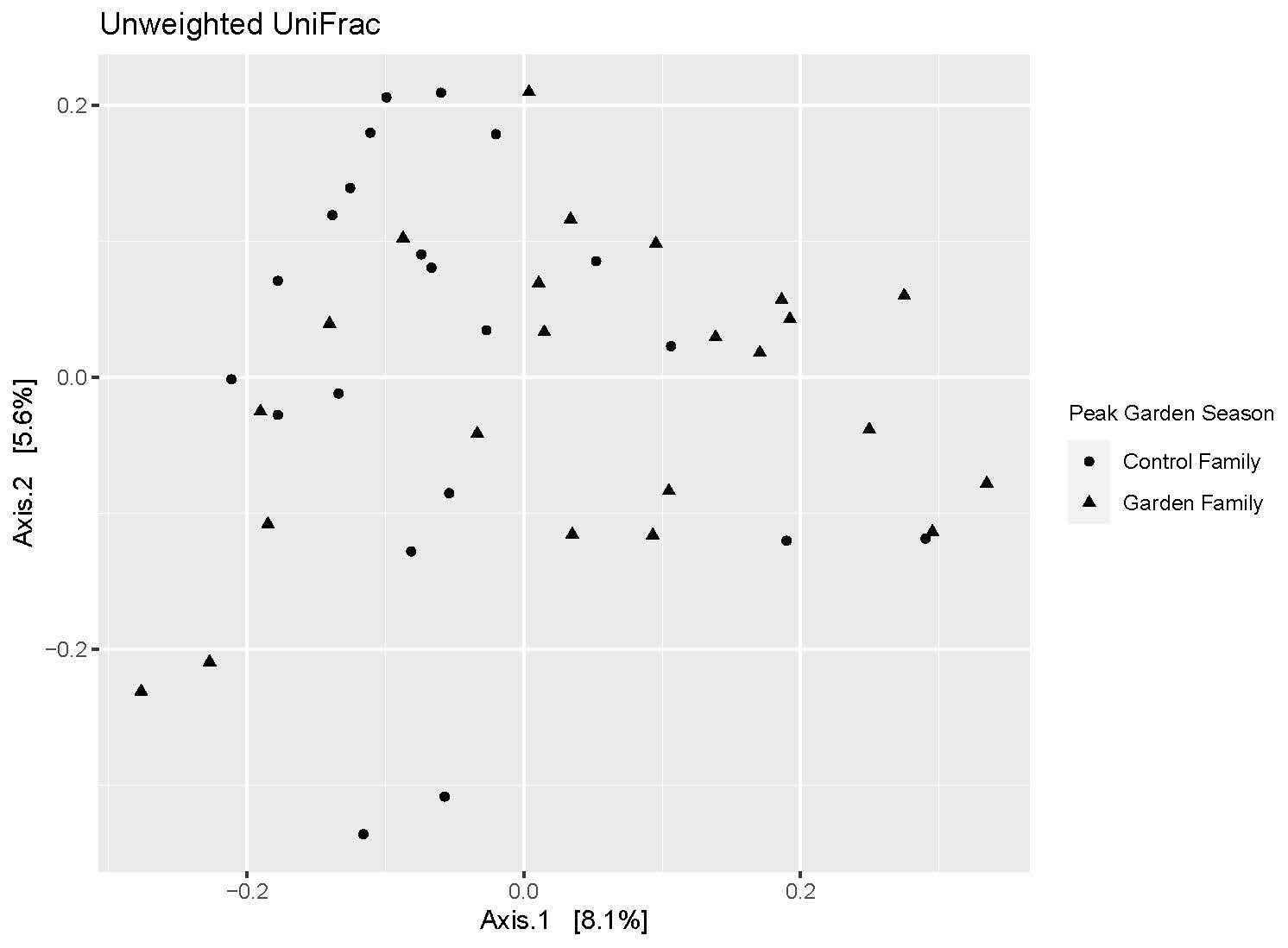


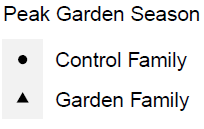


**Supplementary Figure S1** Beta diversity analysis of peak season garden and control families.

LDA Score (log 10)

**Supplementary Figure S2** LEfSe analysis at the genus level between control and garden families at peak gardening season. Microbes shown are significantly greater in gardeners at peak gardening season.

| Genus Enriched in Peak Season Gardeners | | | | | |
| --- | --- | --- | --- | --- | --- |
| Genus^1^ | Control (%) | $\pm$ SEM | Garden (%) | $\pm$SEM | q value^2^ |
| *o_Rhodospirillales_f_uncultured uncultured* | 0.29 | 0.26 | 0.83 | 0.28 | 0.04 |
| *Barnesiella* | 0.62 | 0.28 | 2.04 | 0.49 | 0.04 |
| *f_Christensenellaceae uncultured 1* | 0.00 | 0.00 | 0.00 | 0.00 | 0.04 |
| *_Howardella* | 0.00 | 0.00 | 0.00 | 0.00 | 0.04 |
| *f_Tannerellaceae unknown* | 0.00 | 0.00 | 0.07 | 0.01 | 0.04 |
| *Christensenellaceae R-7 group* | 0.34 | 0.14 | 0.85 | 0.55 | 0.04 |
| *o_Oscillospirales_f_UCG-010 UCG-010* | 0.06 | 0.03 | 0.08 | 0.04 | 0.04 |
| *f_Christensenellaceae uncultured* | 0.00 | 0.00 | 0.00 | 0.00 | 0.04 |
| *o_Coriobacteriales f_uncultured uncultured* | 0.00 | 0.00 | 0.00 | 0.00 | 0.05 |
| *Terrisporobacter* | 0.00 | 0.00 | 0.01 | 0.00 | 0.05 |
| ^1^Average relative abundances (%) of significant genus $\pm$ SEM  ^2^False Discovery Rate was used for adjustment | | | | | |

**Supplementary Table S1** Significance of genus greatest in gardening families at peak garden season compared to peak control.

LDA Score (log 10)

**Supplementary Figure S3** LEfSe analysis at the species level between control and garden families at peak gardening season. Microbes shown are significantly greater in control families when compared to peak season gardening families.

LDA Score (log 10)

**Supplementary Figure S4** LEfSe analysis at the genus level between control and garden families at peak gardening season. Microbes shown are significantly greater in control families when compared to peak season gardening families.

| **Supplementary Table S2** Significance of species greatest in control families compared to gardening families at peak garden season. | | | | | |
| --- | --- | --- | --- | --- | --- |
| Species Enriched in Peak Season Control | | | | | |
| Species^1^ | Control (%) | $\pm$SEM | Garden (%) | $\pm$ SEM | q value^2^ |
| *f_Ruminococcaceae_uncultured uncultured* | 0.02 | 0.00 | 0.00 | 0.00 | 0.01 |
| *Colidextribacter uncultured* | 0.21 | 0.24 | 0.10 | 0.14 | 0.01 |
| *Holdemania uncultured* | 0.02 | 0.00 | 0.01 | 0.00 | 0.02 |
| *Acidaminococcus uncultured* | 0.58 | 0.28 | 0.01 | 0.01 | 0.02 |
| *Ruminococcus_torques_group uncultured* | 1.23 | 0.60 | 0.22 | 0.04 | 0.02 |
| *Flavonifractor uncultured* | 0.16 | 0.03 | 0.08 | 0.02 | 0.02 |
| *Hungatella uncultured* | 0.02 | 0.00 | 0.01 | 0.00 | 0.03 |
| *f_Lachnospiraceae_GCA 900066755 uncultured* | 0.003 | 0.00 | 0.00 | 0.00 | 0.03 |
| *Tyzzerella uncultured* | 0.03 | 0.01 | 0.01 | 0.00 | 0.03 |
| ^1^Average relative abundances (%) of significant species $\pm$ SEM  ^2^False Discovery Rate was used for adjustment | | | | | |

| **Supplementary Table S3** Significance of genus greatest in control families compared to gardening families at peak garden season. | | | | | |
| --- | --- | --- | --- | --- | --- |
| Genus Enriched in Peak Season Control | | | | | |
| Genus^1^ | Control (%) | $\pm$SEM | Garden (%) | $\pm$SEM | q value^2^ |
| *Colidextribacter* | 0.22 | 0.05 | 0.10 | 0.03 | 0.01 |
| *Holdemania* | 0.02 | 0.00 | 0.00 | 0.00 | 0.01 |
| *Acidaminococcus* | 0.58 | 0.28 | 0.01 | 0.01 | 0.02 |
| *Ruminococcus torques group* | 1.39 | 0.60 | 0.42 | 0.12 | 0.02 |
| *Flavonifractor* | 0.16 | 0.03 | 0.08 | 0.02 | 0.02 |
| *Hungatella* | 0.02 | 0.00 | 0.00 | 0.00 | 0.04 |
| *Lachnoclostridium* | 0.42 | 0.11 | 0.32 | 0.15 | 0.04 |
| *Anaerostipes* | 0.90 | 0.27 | 0.24 | 0.03 | 0.05 |
| ^1^Average relative abundances (%) of significant species $\pm$ SEM  ^2^False Discovery Rate was used for adjustment | | | | | |

| **Supplementary Table S4** Gardening survey questionnaire.^1^ | | | | |
| --- | --- | --- | --- | --- |
| Gardening Family | How many years have you been gardening? | During an average week, how much time do you spend growing produce? | How often do you eat the produce you grow? | With whom do you usually garden with? |
| A | 1-2 | Less than 30 minutes a week | Once per week | Children |
| B | 5 | 1-2 hours | 2-3 X month | Spouse and child |
| C | 2-3 | Less than 30 minutes a week | Once per week | Children |
| D | 12 | 1-2 hours | 2-5 X week | Myself |
| E | 7-10 | 5-8 hours | Almost every day | Children |
| F | 5 | 30 minutes to 2 hours | 2-5 X week | Children |
| G | 5-12 | 5-8 hours | 2-5 X week | Children |
| H | 12 | Between 30-59 minutes | 2-3 X month | Spouse and child |
| I | 3-5 | 1-2 hours | 2-5 X week | Children |

^1^Gardening questionnaire was administered at the beginning and at peak gardening season to assess gardener habits. Table is showing gardening habits at peak gardening season.

| **Supplementary Table S5** List of produce grown at peak gardening season.^1^ |
| --- |
| **Vegetables** |
| Cucumbers  Peppers  Banana Peppers  Zucchini  Squash  Green onions  Green beans  Broccoli  Peas  Beans  Carrots  Snap peas  Onions |
| **Fruits** |
| Cherries  Strawberries  Plums  Peaches |
| Grapes  Blackberries  Watermelon  Blueberries  Melon  Raspberries  Tomatoes |
| **Herbs** |
| Mint  Chives  Basil  Catnip  Dill  Cilantro  Stevia |
| ^1^Gardening questionnaire asked participants to list fruits and vegetables typically grown throughout the gardening season. Produce listed above are what gardeners grew at peak gardening season. |


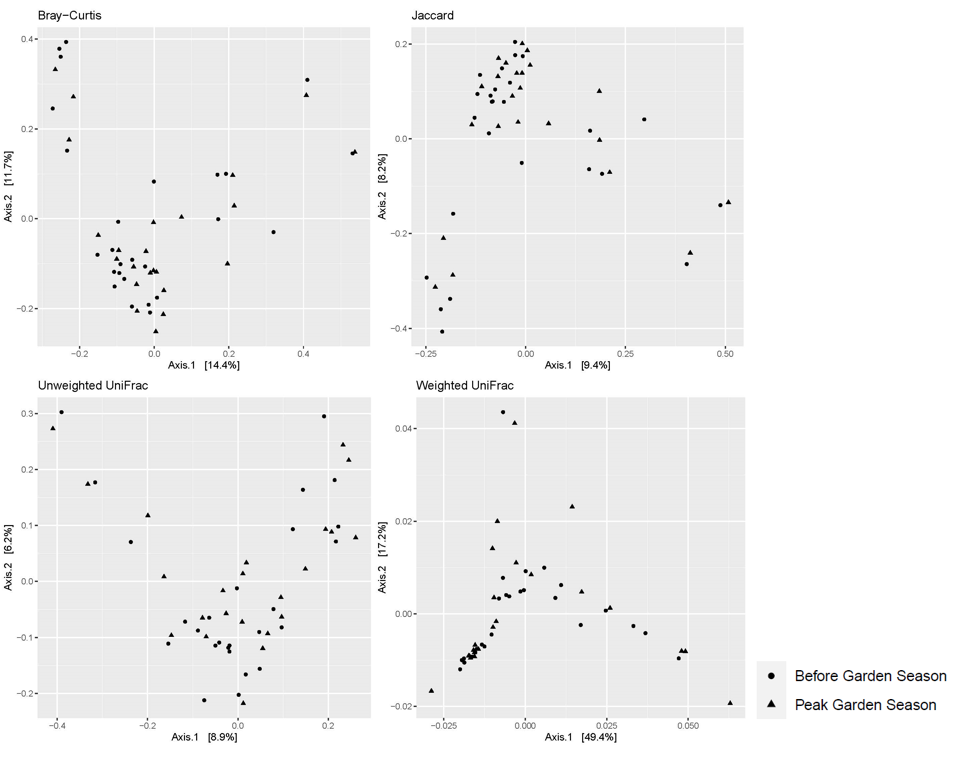


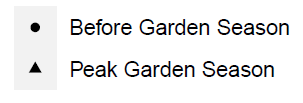


**Supplementary Figure S5** Beta diversity analysis of before garden season and peak garden season within gardening families.
